# Case of Tetanus Cured by Opium

**Published:** 1819-12

**Authors:** E. Sutliffe


FOR THE LONDON MEDICAL AND PHYSICAL JOURNAL.

### *Case of Tetanus cured by Opium.* By E. Sutliffe, Esq.

MUCH has of late been said and written concerning the spinal marrow being implicated in tetanic affections, and very probably with perfect truth, especially when opisthotonos has closed the scene. I well remember a case in Guy's Hospital, under the care of Dr. William Sanders, in 1790 or 91, where this terrific stage of tetanus preceded death; and Dr. S. remarked that, at any future period, under similar circumstances, he should prescribe a bath of solution of opium. Such a decision has never been obliterated from my mind.

Not long since, Mrs. I. of Bartholomew-close, was attacked with spasmodic convulsions about the præcordia, so as to threaten suffocation, ushered in by a severe rigor. After a little enquiry, the symptoms appeared to have their origin from an injured toe, whence the lymphatics were inflamed to the inguen.

Feeling persuaded that the cause was fully ascertained, I commenced with two drachms of tincture of opium, as soon as an interval would admit, and ordered it to be repeated at the expiration of fifteen or twenty minutes. However, the beneficial effects were so obviously decided, that a few hours elapsed before it was repeated, which was then necessary, from a return of the paroxysms; which were however much less violent. I considered the danger not over until the fifth day, from which period the tincture of opium was wholly discontinued. About three ounces were swallowed on this occasion.

*Queen-street; October 5th,* 1819.

P.S.—I presume that Dr. Farre will recognize the case to which I have in a prefatory way referred; to whose friendly assiduity and medical skill, in my own person, recently experienced, I am happy to bear a most grateful testimony.

